# The implementation of routine procedural transvaginal sonography to decrease retained products of conception: a quality improvement initiative

**DOI:** 10.1186/s12905-021-01488-x

**Published:** 2021-10-03

**Authors:** M. Alyssa Larish, E. Claire Jensen, C. Kristin Mara, C. Isabel Green, R. Matthew Hopkins, K. Shannon Laughlin-Tommaso, L. Tatnai Burnett, M. Daniel Breitkopf

**Affiliations:** 1 Department of Obstetrics and Gynecology, Mayo Clinic, 200 1st St. SW, Rochester, MN 55902 USA; 2 Division of Biomedical Statistics and Informatics, Mayo Clinic, 200 1st St. SW, Rochester, MN 55902 USA

**Keywords:** Dilation and suction curettage, Early pregnancy loss, Miscarriage, Pregnancy termination, Products of conception, Ultrasonography

## Abstract

**Background:**

Retained products of conception (POC) following uterine evacuation can lead to adverse sequelae, including hemorrhage, endometritis, intrauterine adhesions, and reoperation. Use of procedural transvaginal sonography (TVUS) in the operating room has been proposed to help decrease retained POC.

**Methods:**

A retrospective review of all first trimester uterine evacuation procedures from 1/2015 to 2/2017 was performed, noting use of transabdominal ultrasonography, retained products of conception, and complications. A practice change was implemented in May 2018, in which routine intra-procedural TVUS use was initiated. A second retrospective chart review was conducted to assess for post-implementation incidence of retained POC, re-operation, and associated complications.

**Results:**

Prior to intra-procedural TVUS implementation, 130 eligible procedures were performed during the specified timeframe, with 9/130 (6.9%) incidence of retained products of conception. TAUS was performed in 59/130 (45.4%) of procedures, and 4/9 (44.4%) of those with retained products. There were eight re-operative procedures in seven patients, and two patients were treated with misoprostol. Complications included hemorrhage, Asherman’s syndrome and endometritis. Following implementation, 95 first trimester procedures were performed with transvaginal sonography, with 0 (0%) cases of retained POC (*p* = 0.01), no incidences of re-operation (*p* = 0.02), and one case of Asherman’s syndrome. TVUS findings led to additional focused suction curettage in 20/95 (21.0%) of procedures. The endometrium was measured on procedure completion in 64 procedures, with a mean thickness of 5.5 mm (1–12 mm).

**Conclusion:**

Implementation of routine TVUS during uterine evacuation may reduce the incidence of retained POC and associated reoperation rates. Further multi-center trials are needed to confirm this finding.

**Supplementary Information:**

The online version contains supplementary material available at 10.1186/s12905-021-01488-x.

## Background

First trimester uterine evacuation via medical or surgical means is relatively common, performed for either pregnancy termination or evacuation following pregnancy loss [1, 2]. Conventional management options include medical treatment with misoprostol and or mifepristone, manual vacuum aspiration, and surgical evacuation with dilatation and suction curettage (D&C).

Due to the limitations of medical management, including treatment failure, many women elect for primary surgical evacuation of the uterine cavity via suction D&C, also known as Electric Vacuum Aspiration (EVA) [3]. Inherent risks of this procedure include hemorrhage, infection, perforation, intrauterine adhesion formation, retained products of conception (POC), with overall complication rates ranging from 0.9 to 8.4% [4–6]. In studies with adequate follow-up, the incidence of retained POC following these procedures ranges from 1.5 to 3.7%, with an average of 4% of patients requiring reoperation, either to remove retained products, or treat hemorrhage or other complications [6–8]. Post-operatively, retained products of conception may lead to endometritis, intrauterine adhesions and ultimately impairment of fertility [9].

Intraoperatively, consideration should be given to balance the risk of retained products of conception with the risk of intrauterine adhesions from overly aggressive suction curettage. To address this, the use of ultrasonography during the uterine evacuation was first described for difficult dilation and evacuation procedures in the 1980’s [10, 11]. We noted an above average rate of retained products of conception at our institution, despite frequent use of transabdominal ultrasound in nearly half of cases, which was confirmed on retrospective chart review. Literature review demonstrated efficacy in the routine use of transvaginal ultrasound following uterine evacuation to assist in decreasing the rate of retained products of conception [6]. Additionally, evidence of the superiority of transvaginal over transabdominal sonography in assessing endometrial pathology and thickness has been clearly demonstrated in the postmenopausal patients presenting with abnormal bleeding, and is indeed the only ultrasonography route suggested by the American College of Obstetrics and Gynecology (ACOG) for endometrial assessment [12, 13]. Given this information, we chose to study the impact of a quality improvement practice change to the use of intraoperative transvaginal sonography during the dilation and suction curettage procedure on our incidence of retained products of conception.

## Methods

Following formal protocol review, this study was deemed Institutional Review Board (IRB) exempt at Mayo Clinic as it pertained to quality and process improvement, and therefore informed consent was not required of participants. The study was completed without external funding. All methods were carried out in accordance with relevant guidelines and regulations. A comprehensive retrospective chart review of all first trimester uterine evacuation procedures performed from January 2015 through February 2017 was completed. Records reviewed included clinic notes, emergency room records, operative notes, pathology reports, and all communications in the electronic medical record. Eligible patients included patients over 18 years of age with a gestation of age less than or equal to 14 weeks by ultrasound and completion of a dilation and curettage procedure under anesthesia. It is our practice to counsel patients regarding the three options of expectant management, medical management, or surgical management to enact first trimester uterine evacuation. Patients whom elected to undergo, and whom completed expectant or medically managed uterine evacuation were excluded from the study. Special notation was taken on the baseline (pre-intervention) use of intraprocedural transabdominal ultrasonography, overall complications, reoperation, and cases of retained products of conception. The procedures were typically performed by junior residents supervised by faculty. Standardized departmental practice was followed, including the administration of 400 ug misoprostol upon arrival to the pre-operative area for the indication of cervical ripening, use of plastic suction curettage instrumentation, and depending on surgeon preference, confirmation of complete uterine evacuation via the tactile feedback of gentle curettage at procedural completion. No evacuation of uterine contents was performed with sharp curettage, nor was aggressive sharp curettage permitted. Anesthetic ranged from sedation to monitored anesthesia care (MAC) to general anesthesia. Retained products of conception were defined as symptoms (bleeding, cramping, or infection) leading to a workup which revealed suspicious ultrasonographic findings (heterogeneous, thickened endometrium with focal Doppler flow), ultimately mandating intervention with medical or surgical treatment. Pathologic confirmation of retained products of conception for patients was requested at the discretion of the faculty if visual confirmation or villi were unable to be confidently identified in the operating room, as were serum measurements of beta-human chorionic gonadotropin (b-HCG). Asymptomatic patients with an incidental discovery of postoperative endometrial thickening or focal Doppler flow which did not require intervention were not considered to have retained products of conception.

**Fig. 1:**
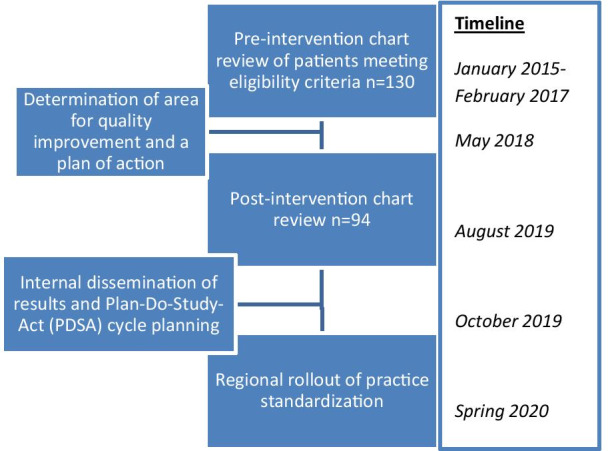
Quality improvement process workflow and timeline. An overview of our methodology and study timeframe

In May of 2018, a practice change was implemented involving routine use of intra-procedural transvaginal sonography during first trimester dilation and suction curettage procedures occurring at less than 14 weeks gestation by ultrasound, and documentation of findings in the operative note. Surgeons assessed the appearance of the endometrial stripe and were encouraged to document endometrial thickness (ET) for study purposes; however, they were not required to target a specific thickness threshold for intervention with additional suction curettage. Transvaginal ultrasound was performed at the conclusion of the procedure, and any subsequent targeted suction curettage based on ultrasonographic findings was noted in the operative report. If this focal re-evacuation was performed, a final transvaginal ultrasound was performed again at case completion. Color doppler flow was utilized at the discretion of the provider to further characterize any focal thickening. The protocol for this is detailed in Additional file 1: Appendix S1. In August 2019, a second retrospective chart review was performed to assess for post-implementation compliance, incidence of retained POC, and complications including reoperation. Of note, manual vacuum aspiration (MVA) was not offered prior to the study timeframe, but was incorporated into the practice in August 2019, outside of the timeframe for this study. The quality improvement study design and timeline is summarized in Fig. 1. Comparisons of demographics and complications between the two time periods were made using Fisher’s exact tests, with *p* < 0.05 considered statistically significant. Frequencies and percentages are used to describe the results.

## Results

Baseline demographics of pre- and post-intervention groups are summarized in Table 1. No differences in maternal age, gravidity, parity, cesarean delivery, gestational age, previous D&C, misoprostol and/or mifepristone administration > 24 h pre-pre procedure, molar pregnancy, uterine infection at presentation were noted. There was a difference in the proportion of cases diagnosed with missed abortion (84.6% vs. 71.6%) versus incomplete abortion (10% vs. 27.4%) pre- and post-intervention, respectively.

**Table 1:** Patient demographics and clinical characteristics before and after ultrasound intervention

	Pre intervention N = 130	Post intervention N = 95	*p* value
Age at time of procedure (median, range)	32 (18–43)	33 (19–48)	0.64
Gravidity (median, range)	2 (1–8)	3 (1–10)	0.10
Parity (median, range)	1 (0–6)	1 (0–6)	0.19
Previous cesarean (N, %)	17, 13.1	13, 14.0	0.80
Previous Dilation and Curettage procedures (N, %)	29, 22.3	15, 16.1	0.30
Previous medical abortion or medical management of missed abortion (N, %)	23, 17.7	21, 22.3	0.40
Gestational Age in weeks at time of procedure by US (median, range)	7 (4–12)	7 (4–14)	0.67
Uterine Infection at presentation (N, %)	1, 0.8	2, 2.1	0.39
Missed Abortion^a^ (N, %)	110, 84.6	68, 71.6	0.018
Incomplete Abortion^a^ (N, %)	13, 10	26, 27.4	< 0.001
Molar Pregnancy^a^ (N)	2	3	0.70

^a^Characterizations of type of abortion or type of pregnancy are not mutually exclusive, and patients may be classified into more than one group

Prior to intra-procedural TVUS implementation, 130 eligible first trimester uterine evacuation procedures were performed between January 1, 2015 and February 28, 2017. Patient characteristics and demographics are summarized in Table 1. The pre-intervention incidence of retained products of conception was 9/130 (6.9%), diagnosed a median of 46 days (range 3–80 days) postoperatively. Of the nine patients with retained products, five had a history of previous cesarean, three had a history of previous dilation and curettage, no patients had a history of myomectomy, and one pregnancy was achieved via in-vitro fertilization. Intraprocedural transabdominal ultrasonography had been performed to assess for complete uterine evacuation in 59 of the 130 procedures (45.4%), and in four of nine (44.4%) of those with retained products. Of the nine patients with retained products, two were treated with misoprostol, and seven underwent a total of eight operative procedures to resect retained products. One of the seven patients undergoing reoperation, had two subsequent procedures due to inadequate resection of retained products at the time of first reoperation. Resection of retained products was performed hysteroscopically in five cases to achieve a targeted resection in patients with suspicious focal ultrasonography findings, such as regional thickening with abnormal color doppler flow. Of the seven patients undergoing re-operation, pathologic confirmation was requested in five patients, with findings of definitive chorionic villi in four, and a degraded sample in one with abnormal serum human gonadotropin level. The remainder was confirmed visually with hysteroscopy or via floating of villi. B-hCG levels were assessed in five patients, and levels ranged from 15 to 3794 milli-international units/ milliliter (mIU/mL). Complications following initial procedures occurred in 3 patients: one event of hemorrhage, one case of Asherman’s syndrome confirmed on hysteroscopy, and one clinically diagnosed case of endometritis.

Following implementation of routine transvaginal sonography, 95 first trimester procedures were performed, with 0 cases of retained POC (*p* = 0.01), no incidences of re-operation (*p* = 0.02), and one case of Asherman’s syndrome (confirmed hysteroscopically) in a patient with multiple D&Cs prior to the study timeframe. There was one case of intraoperative asystole attributed to underlying congenital cardiopulmonary disease. Transvaginal ultrasonography led to additional focused suction curettage of the areas suspected to contain residual products of conception (non-uniform, thickened, or Doppler flow within the endometrium) in 20/95 (21.1%) of procedures. While transvaginal sonography was performed during all procedures, the final endometrial thickness was documented in the operative note at procedural completion in 64/95 (67.4%) procedures, with a mean thickness of 5.5 mm (range 1–12 mm). These results are summarized in Table 2.

**Table 2:** Comparison of complications pre and post intervention

Complication	Pre-intervention group (*n* = 130) (*n* (%))	Post intervention group (*n* = 95) (*n* (%))	*P*
Retained products of conception	9 (6.9)	0 (0.0)	< 0.01
Re-operative Procedures	8 (6.15)	0 (0.0)	0.02
Endometritis	1 (0.7)	0(0.0)	NS
Uterine perforation	0 (0.0)	0 (0.0)	NS
Hemorrhage	3 (2.3)	0 (0.0)	NS
Asherman’s Syndrome	1 (1.5)	1 (1.1)	NS

## Discussion

This study highlights the successful implementation of a quality improvement process and protocol using transvaginal ultrasound during dilation and suction curettage to decrease retained products of conception and associated re-operation rates in an academic teaching facility. We also observed a trend toward a decrease in associated complications such as hemorrhage and endometritis; however this did not reach statistical significance. Importantly, we did not encounter an increased incidence of quality improvement balancing measures (defined as when a positive change in one part of a system adversely impacts another), or in sequelae such as Asherman’s syndrome or uterine perforation following implementation.

Strengths of our study include the standardized definition of retained products of conception to decrease observer bias. Retained POC were defined by both symptoms which led to workup revealing ultrasonographic findings meriting medical or surgical intervention (heterogeneous and thickened endometrium with Doppler flow), and confirmed by pathologic and /or visual analysis of the retained products. Importantly, the finding of a thickened and irregular endometrium with low-impedance Doppler flow post procedure can be a normal variant in up to 32% of women at 1 week post-procedure [14]. Therefore, a comprehensive clinical assessment should be used in each case to determine the probability of retained products of conception before committing to intervention. Last, a major strength of our study was that our patient population in Olmstead County exhibited near universal follow up for post-operative miscarriage check in appointments, making the determination of both procedural complications and re-treatment much more reliable.

Limitations of our study include the possibility of a Hawthorne effect in which surgeons alter their procedural performance and attention to detail upon learning their outcomes are being studied. We also attribute the increased diagnosis of incomplete abortions following implementation to improved documentation regarding case details such as preoperative bleeding or cervical dilation, a result of both awareness of study initiation among surgeons, as well as the transition to a new electronic medical record and diagnostic codes concurrent with the post-implementation phase. Other limitations include the quality improvement based methodology, which allows for uniform implementation of the intervention without enrollment in a prospective cohort or randomized controlled trial. Fortunately, this methodology allows continuation of the quality improvement process via monitoring of retained product of conception rates going forward. Additionally, our study had a small sample and event size, prohibiting statistical adjustment for characteristic differences. However, in spite of these limitations, overt statistical significance was found. Last, we also acknowledge the evidence basis for the safety of manual vacuum aspiration, which was temporarily not performed at our institution during this timeframe. Indeed, previous authors have demonstrated similarly low complication rates with MVA when compared to EVA [15–17], suggesting that inferences of the benefit of transvaginal ultrasonography in this population can be made as well.

While we were able to obtain a significant reduction in retained products of conception, the pre-intervention incidence of retained POC in the current study was slightly higher than reported in the literature, which may have biased our results toward significance. This may have been representative of several factors, including near complete patient follow up to detect all cases, as well as the complexity of some patients referred to a tertiary referral center. We are encouraged that our findings are congruent with the previous report by Debby et al., who noted a decreased incidence in retained products of conception while curetting to a goal of an endometrial thickness of less than or equal to 8 mm [6]. Importantly, our study differed in two ways: first in the quality improvement based design with an emphasis on a process based improvement, and second that we allowed surgeon autonomy by not mandating an arbitrary cut off of endometrial thickness. Wong et al. found that diagnosing retained products of conception based on an endometrial thickness greater than 8 mm has been shown to have a limited specificity of approximately 80% [18]. Therefore, we advocated using the surgeon’s clinical experience in interpreting the appearance of a patient’s endometrium on ultrasound, resulting in no cases of retained products of conception.

Our findings suggest that transvaginal sonography can be used as an effective safety check to confirm complete uterine evacuation following dilation and suction curettage procedures. The addition to the procedure adds no surgical risk to the patient, but does provide reassurance to both the surgeon and the patient. While we did not bill for our use of bedside sonography during this quality improvement study, this could be done at the discretion of providers. Last, training residents in the use of transvaginal sonography is an important skillset for future independent practice, and scanning in this supervised setting augments their experience. While our study focused on first trimester procedures, additional studies should be done to evaluate the use of transvaginal sonography in second trimester dilation and evacuation procedures and in patients with Mullerian Anomalies. Previous studies have suggested curetting to a goal endometrial thickness to be assured of uterine evacuation [6]; however we propose that larger multi-center studies are needed to validate the safety of both this objective cutoff-based and our subjective approaches.

## Conclusions

We found that a quality improvement based implementation of routine transvaginal ultrasonography during first trimester uterine evacuation appears to reduce the incidence of both retained products of conception and associated re-operation rates at an academic teaching facility, without increasing other procedural complications.

## Supplementary Information

**Additional file 1: Appendix S1**. Transvaginal ultrasonography after D&C for early pregnancy evacuation protocol

## Data Availability

The datasets used and/or analysed during the current study are available from the corresponding author on reasonable request.

## Abbreviations

POC: Retained products of conception
TVUS: Transvaginal Ultrasonography
D&C: Dilation and Curettage
EVA: Electric Vacuum Aspiration
ACOG: American College of Obstetrics and Gynecology
IRB: Institutional Review Board
HCG: Human chorionic gonadotropin
MVA: Manual vacuum aspiration
ET: Endometrial thickness
MAC: Monitored anesthesia care
mIU/mL: Milli-international units/ milliliter
