# Supplementary material for: The implementation of routine procedural transvaginal sonography to decrease retained products of conception: a quality improvement initiative
Source: BMC Womens Health. 2021 Oct 3;21:347. doi: 10.1186/s12905-021-01488-x (PMC8489076; doi:10.1186/s12905-021-01488-x)
Supplement: Supplementary file 1 — Additional file 1: Appendix S1. Transvaginal ultrasonography after D&C for early pregnancy evacuation protocol [file 12905_2021_1488_MOESM1_ESM.docx]

*The implementation of routine procedural transvaginal sonography to decrease retained products of conception: A quality improvement initiative*

***LARISH Alyssa M MD^1^, JENSEN Claire E MD^1^, MARA Kristin C MS ^2^, GREEN Isabel C MD^1^, HOPKINS Matthew R MD^1^, LAUGHLIN-TOMMASO Shannon K MD^1^, BURNETT Tatnai L MD^1^, BREITKOPF Daniel M MD^1^***

**Appendix S1: Transvaginal ultrasonography after D&C for early pregnancy evacuation protocol**

We recommend the following procedure following all first trimester (≤14 weeks by ultrasonography) pregnancy related uterine evacuation procedures:

1. Perform uterine evacuation procedure according to standard of practice
2. Upon procedural completion, perform an exam of the entirety of the uterine lining using transvaginal sonography, looking for areas of thickened stripe or vascularity with doppler flow. Probe covers should be clean but do not need to be sterile.

Measurements should include the endometrial stripe thickness. Save images.

*If re-curettage is performed based on the ultrasound findings, re-ultrasound at completion and document re-curettage*.

1. Documentation: (brief operative note and operative note)- note findings on ultrasound, including final endometrial stripe thickness.
2. Discuss ultra-sonographic findings with patient in clinic, or when awakened from anesthesia.

*******************************************************************************************************
